# Adapting the Surveillance Platform for Enteric and Respiratory Infectious Organisms at United States Veterans Affairs Medical Centers (SUPERNOVA) for COVID-19 Among Hospitalized Adults: Surveillance Protocol

**DOI:** 10.3389/fpubh.2021.739076

**Published:** 2021-10-29

**Authors:** Elissa Meites, Kristina L. Bajema, Anita Kambhampati, Mila Prill, Vincent C. Marconi, Sheldon T. Brown, Maria C. Rodriguez-Barradas, David O. Beenhouwer, Mark Holodniy, Cynthia Lucero-Obusan, Cristina Cardemil, Jordan Cates, Diya Surie

**Affiliations:** ^1^National Center for Immunization and Respiratory Diseases, Centers for Disease Control and Prevention, Atlanta, GA, United States; ^2^Atlanta VA Medical Center, Atlanta, GA, United States; ^3^Department of Medicine, Emory University School of Medicine, Atlanta, GA, United States; ^4^Department of Global Health, Rollins School of Public Health, Emory University, Atlanta, GA, United States; ^5^James J. Peters VA Medical Center, Bronx, NY, United States; ^6^Department of Medicine, Icahn School of Medicine at Mount Sinai, New York, NY, United States; ^7^Michael E. DeBakey VA Medical Center, Houston, TX, United States; ^8^Department of Medicine, Baylor College of Medicine, Houston, TX, United States; ^9^VA Greater Los Angeles Healthcare System, Los Angeles, CA, United States; ^10^Department of Medicine, David Geffen School of Medicine at UCLA, Los Angeles, CA, United States; ^11^VA Palo Alto Health Care System, Palo Alto, CA, United States; ^12^Public Health Surveillance and Research, Department of Veterans Affairs, Washington, DC, United States; ^13^Department of Medicine, Stanford University, Stanford, CA, United States

**Keywords:** surveillance, public health, COVID-19, Veterans health, epidemiology, research methods

## Abstract

**Introduction:** Early in the COVID-19 pandemic, the Centers for Disease Control and Prevention (CDC) rapidly initiated COVID-19 surveillance by leveraging existing hospital networks to assess disease burden among hospitalized inpatients and inform prevention efforts.

**Materials and Methods:** The Surveillance Platform for Enteric and Respiratory Infectious Organisms at Veterans Affairs Medical Centers (SUPERNOVA) is a network of five United States Veterans Affairs Medical Centers which serves nearly 400,000 Veterans annually and conducts laboratory-based passive and active monitoring for pathogens associated with acute gastroenteritis and acute respiratory illness among hospitalized Veterans. This paper presents surveillance methods for adapting the SUPERNOVA surveillance platform to prospectively evaluate COVID-19 epidemiology during a public health emergency, including detecting, characterizing, and monitoring patients with and without COVID-19 beginning in March 2020. To allow for case-control analyses, patients with COVID-19 and patients with non-COVID-19 acute respiratory illness were included.

**Results:** SUPERNOVA included 1,235 participants with COVID-19 and 707 participants with other acute respiratory illnesses hospitalized during February through December 2020. Most participants were male (93.1%), with a median age of 70 years, and 45.8% non-Hispanic Black and 32.6% non-Hispanic White. Among those with COVID-19, 28.2% were transferred to an intensive care unit, 9.4% received invasive mechanical ventilation, and 13.9% died. Compared with controls, after adjusting for age, sex, and race/ethnicity, COVID-19 case-patients had significantly higher risk of mortality, respiratory failure, and invasive mechanical ventilation, and longer hospital stays.

**Discussion:** Strengths of the SUPERNOVA platform for COVID-19 surveillance include the ability to collect and integrate multiple types of data, including clinical and illness outcome information, and SARS-CoV-2 laboratory test results from respiratory and serum specimens. Analysis of data from this platform also enables formal comparisons of participants with and without COVID-19. Surveillance data collected during a public health emergency from this key U.S. population of Veterans will be useful for epidemiologic investigations of COVID-19 spectrum of disease, underlying medical conditions, virus variants, and vaccine effectiveness, according to public health priorities and needs.

## Introduction

In response to the emergence of a novel coronavirus, severe acute respiratory syndrome coronavirus 2 (SARS-CoV-2), the Centers for Disease Control and Prevention (CDC) activated its Emergency Operations Center on January 21, 2020, to offer operational support and public health expertise (1). By February 2020, CDC had initiated COVID-19 surveillance by leveraging existing systems, including the Surveillance Platform for Enteric and Respiratory Infectious Organisms at the VA (SUPERNOVA). SUPERNOVA is a network of VA Medical Centers (VAMC) conducting laboratory-based passive and active monitoring for pathogens associated with acute gastroenteritis and acute respiratory illness among hospitalized adult Veterans in the United States.

Over 9 million Veterans—people who have served as military, naval, air, and other uniformed service members in the United States—currently receive health care from the Veterans Health Administration of the Department of Veterans Affairs (VA), an integrated health care delivery network providing comprehensive health services to eligible Veterans through medical centers and community-based outpatient clinics across the United States (2, 3). Within the VA network, sites use a comprehensive electronic health record system, Veterans Health Information Systems and Technology Architecture (VistA), and Computerized Patient Record System (CPRS), with existing functionalities that support data collection for clinical and research purposes (4). Patients served by the VA are predominantly male (88%), older, and with a higher illness burden compared to civilians (5, 6). Veterans who are of Black/African-American race or Hispanic ethnicity may seek care within the VA system more commonly than Veterans of other racial/ethnic groups (7).

Data from Veterans can provide key insights into the impact of COVID-19 in the United States. During the COVID-19 pandemic, people who are male, aged >65 years, or of Black race were identified as being disproportionately affected by severe illness caused by SARS-CoV-2 infection, (8, 9) supporting the importance of studying this novel disease among VA patients. In addition, certain underlying medical conditions common among Veterans such as chronic lung diseases, diabetes, and heart conditions (5), are known to increase the risk of severe COVID-19 illness (10). Early COVID-19 research using large national datasets from hospitalized U.S. Veterans have noted increased risk for in-hospital complications with COVID-19 compared to influenza (11); identified associations between excess burden of COVID-19 and race/ethnicity (12); evaluated risk factors for hospitalization, mechanical ventilation, or death (13); assessed critical care strain (14); and described readmissions and deaths within 60 days after initial hospital discharge (15).

Although disease dynamics among VA patients are not necessarily comparable to those among members of the general U.S. population, examination of populations at increased risk for severe disease is relevant to understanding the full range of COVID-19 effects. Prospective surveillance at sentinel sites, as in SUPERNOVA, while not nationally representative, allows for collection of granular data, such as results of laboratory testing on clinical specimens, that can be challenging to capture in larger analyses of administrative data. In this paper, we present surveillance methods for adapting the SUPERNOVA surveillance platform to prospectively evaluate COVID-19 epidemiology during a public health emergency, including detecting, characterizing, and monitoring patients with and without COVID-19.

## Materials and Methods

**Figure 1:**
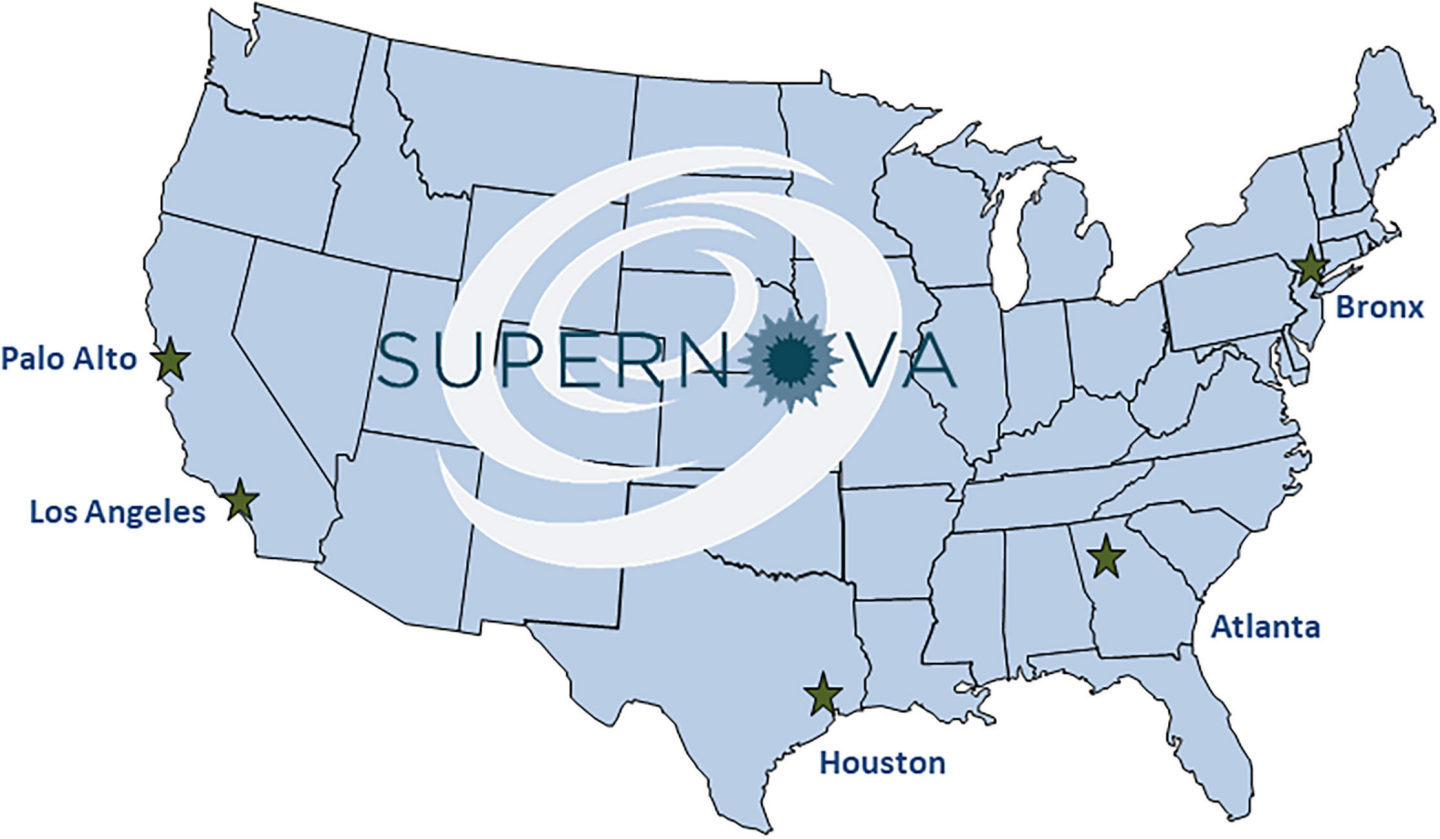
Map of five sentinel sites conducting COVID-19 surveillance—Surveillance Platform for Enteric and Respiratory Infectious Organisms at Veterans Affairs Medical Centers (SUPERNOVA), United States, 2020–2021.

SUPERNOVA is a sentinel network of five participating VAMCs located in four U.S. states (Figure 1), collectively serving an estimated patient population of 400,000 Veterans annually (Table 1). Surveillance is conducted among Veterans aged ≥18 years who seek care at a participating VAMC.

**Table 1:** Selected characteristics of participating sentinel sites—Surveillance Platform for Enteric and Respiratory Infectious Organisms at Veterans Affairs Medical Centers (SUPERNOVA).

**Site name and location**	**Size**	**Description**	**Estimated population served annually**
Atlanta VA Medical Center, **Atlanta, GA, USA**	405 beds	Tertiary care facility (teaching hospital) with 273 hospital beds, CLC with 120 beds, Psychosocial Rehabilitation Treatment Program with 12 beds; seven CBOCs	120,000 Veterans in northeastern Georgia (GA)
James J. Peters VA Medical Center, **Bronx, NY, USA**	431 beds	Tertiary care facility (teaching hospital) with 311 hospital beds, CLC with 120 beds; three CBOCs	24,000 Veterans in the New York City metropolitan region in New York (NY)
Michael E. DeBakey VA Medical Center, **Houston, TX, USA**	531 beds	Tertiary care facility (teaching hospital) with 350 acute care beds, Spinal Cord Injury Center with 40 beds, CLC with 141 beds; 10 CBOCs	113,000 Veterans in southeastern Texas (TX)
VA Greater Los Angeles Health System, **Los Angeles, CA, USA**	945 beds	Tertiary care facility (teaching hospital), CLC, community care center for homeless Veterans; eight CBOCs	82,000 Veterans in southern California (CA)
VA Palo Alto Health Care System, **Palo Alto, CA, USA**	800 beds	Three tertiary care facilities (teaching hospital), homeless domiciliary with 100 beds, three CLCs; seven CBOCs	67,000 Veterans in northern California (CA)

*Community Living Centers (CLCs) provide extended care rehabilitation and general long-term care; Community-Based Outpatient Clinics (CBOCs) include outpatient primary care*.

### Surveillance for Acute Gastroenteritis and Acute Respiratory Illness

**Figure 2:**
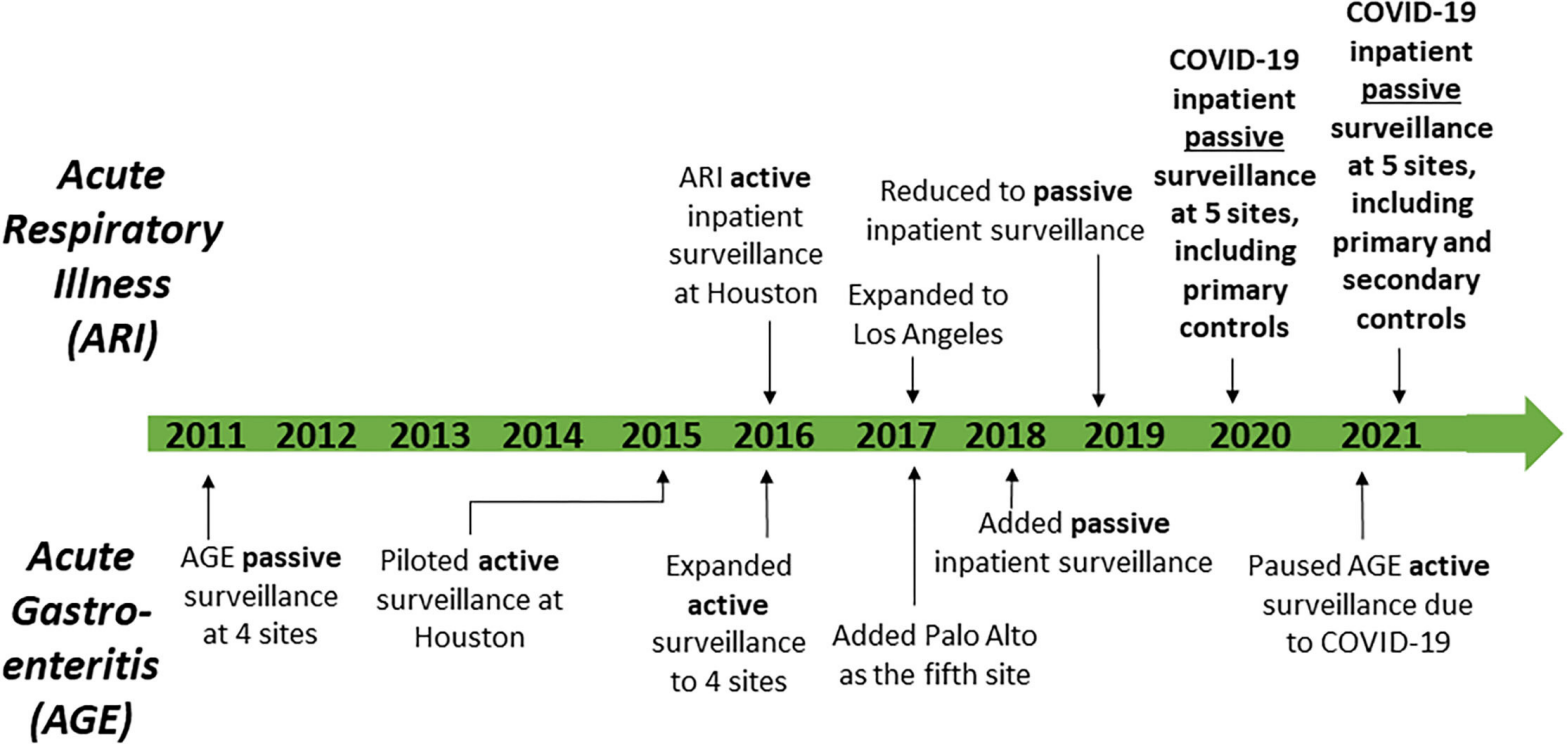
Timeline of surveillance activities—Surveillance Platform for Enteric and Respiratory Infectious Organisms at Veterans Affairs Medical Centers (SUPERNOVA), 2011–2021.

Before 2020, primary objectives for the SUPERNOVA network included surveillance to estimate the incidence of norovirus and other causes of acute gastroenteritis in inpatient and outpatient settings, and active and passive surveillance to determine the inpatient burden of adult respiratory syncytial virus, influenza viruses, and other viruses that cause acute respiratory illness (Figure 2). Acute gastroenteritis surveillance using stool specimens ordered for clinician-directed diagnostic testing began at four sites in 2011, and expanded in 2015 to include medical chart review and collection of saliva and serum specimens, with a fifth site (Palo Alto) added in 2017 (16, 17). Acute respiratory illness surveillance with laboratory confirmation began as a pilot at one site (Houston) in 2016, with a second site (Los Angeles) added in 2017. Acute respiratory illness surveillance included: (1) identification of patients who had been admitted with a diagnosis consistent with acute respiratory illness and/or had received clinician-ordered viral respiratory diagnostic testing; (2) medical record reviews and chart abstractions to obtain demographic and clinical information including clinical course; and (3) collection of residual respiratory and serum specimens (18). By 2019–2020, surveillance for acute gastroenteritis and acute respiratory illness was including ~900 and ~1,600 patients, respectively.

### Adaptation for COVID-19 Surveillance

Beginning in March 2020, SUPERNOVA shifted focus to expand acute respiratory illness surveillance to prospective inpatient surveillance with a focus on COVID-19 at all five participating VAMC sites, while pausing surveillance for acute gastroenteritis. Objectives included understanding characteristics of patients with COVID-19 and the spectrum of disease severity, COVID-19 hospitalization rates, underlying medical conditions that increase risk of severe outcomes, virus variants, immune responses, multisystem inflammatory syndrome in adults, and health care use among patients with COVID-19. Additionally, given inclusion of case and control groups, this surveillance platform was positioned to assess COVID-19 vaccine effectiveness among hospitalized Veterans.

### Identification of Patients With and Without COVID-19

**Figure 3:**
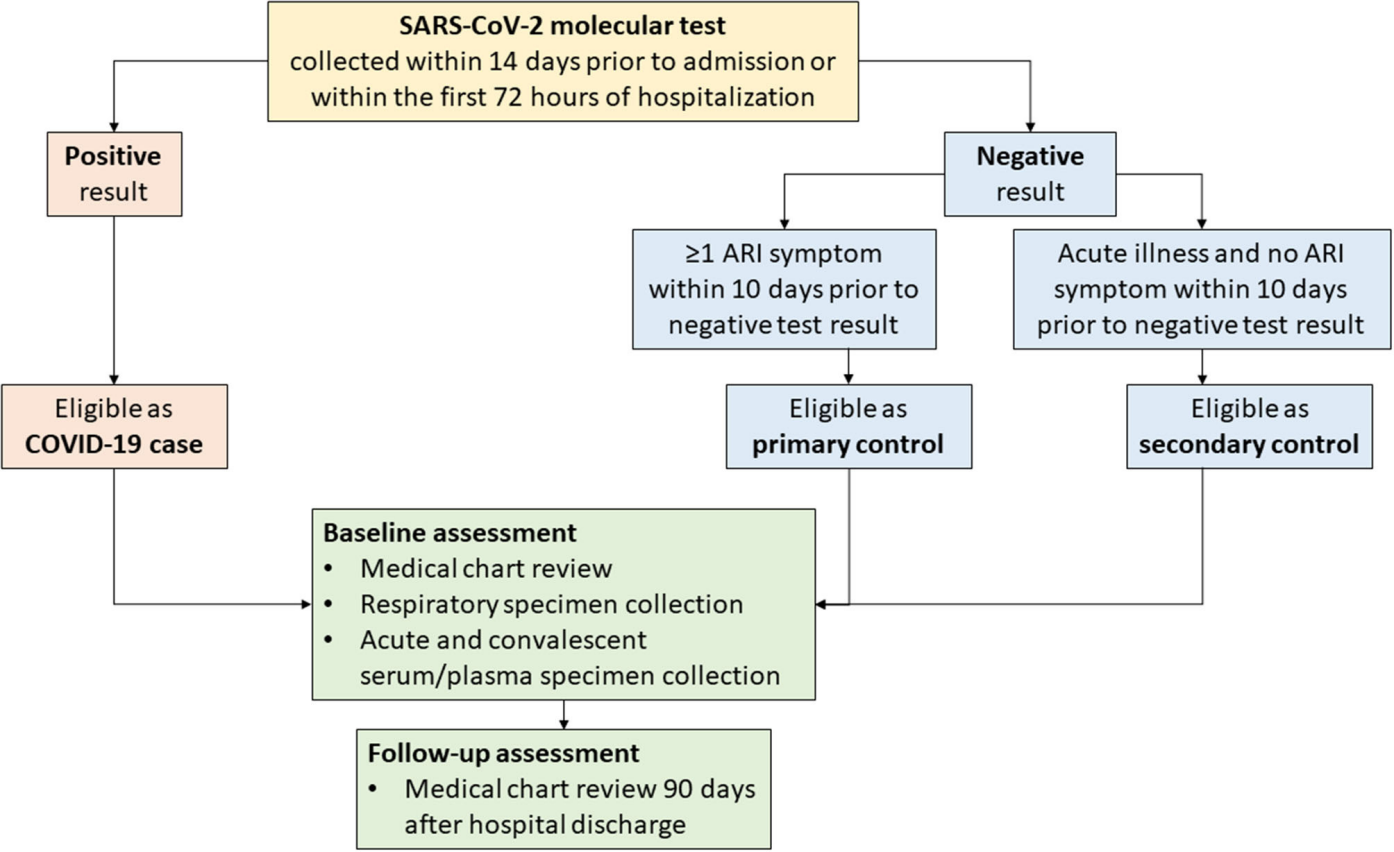
Flow diagram for COVID-19 surveillance data collection—Surveillance Platform for Enteric and Respiratory Infectious Organisms at Veterans Affairs Medical Centers (SUPERNOVA), 2021. ARI, acute respiratory illness.

Beginning in February 2020, clinician-directed testing and then universal inpatient screening at hospital admission for SARS-CoV-2 were implemented at the five VAMC sites. Patients with COVID-19 (i.e., cases) were eligible for inclusion in SUPERNOVA if they had a positive molecular SARS-CoV-2 test result within the first 72 hours of hospitalization (Figure 3). Cases were considered symptomatic if they had ≥1 COVID-19-like signs or symptoms documented in their medical records (e.g., admission history and physical examination, or emergency department note)^1^.

To allow for case-control analyses, for each case, 1–2 inpatients without COVID-19 (i.e., controls) were targeted for inclusion in SUPERNOVA (Figure 3). In keeping with existing surveillance procedures for acute respiratory illness within SUPERNOVA, controls were identified from among other patients with respiratory symptoms. Patients were eligible for inclusion as a non-COVID-19 control if they had both a negative SARS-CoV-2 molecular test result (reverse transcription-polymerase chain reaction [RT-PCR] or isothermal nucleic acid amplification test [NAAT]) within 14 days prior to admission or within the first 72 hours of hospitalization, and, if they presented with ≥1 respiratory symptom^2^ (primary controls) at admission. An additional set of controls without respiratory symptoms was included beginning in early 2021. Secondary controls, defined as persons admitted for a non-respiratory acute illness (e.g., chest pain, hypotension) with a negative test result for SARS-CoV-2 and without respiratory symptoms, were also included. Primary or secondary controls who later had a positive SARS-CoV-2 antigen or molecular test result during hospitalization would become ineligible as controls; however, controls could have a positive test result for a different pathogen (e.g., influenza). To ensure similar participation dates, controls were identified daily according to hospital admission dates falling within 1–14 days of COVID-19 case admission dates.

### Medical Record Review

VAMC electronic health records (EHR) of participants with and without COVID-19 were reviewed. Using standardized case report forms, information was collected on patient demographic and clinical characteristics, including SARS-CoV-2 and other viral pathogen test results; history of vaccination against COVID-19 (including state immunization registry data, where available), influenza, and pneumococcal disease; history of present illness and clinical presentation with vital signs and laboratory values upon admission; past medical history including underlying medical conditions; imaging and interventions including intensive care unit admission and mechanical ventilation; treatments provided during hospitalization (e.g., anti-inflammatory agents, anticoagulants, antivirals, vasopressors, hemodialysis, and other therapies); hospital complications and discharge diagnoses using ICD-10 codes; advance directive code status, and disposition (including deaths). Medical records of both cases and controls were reviewed again 90 days after hospital discharge to assess outcomes including clinical and functional status, and additional health care use.

### Clinical Specimens

Residual clinical respiratory swab specimens and serum or plasma specimens were collected from both COVID-19 case-patients and controls where possible. Residual respiratory specimens were stored at −70°C before shipment to CDC for testing using CDC's 2019-Novel Coronavirus Real-Time RT-PCR Diagnostic Panel (19). Residual serum or plasma specimens (1–2 mL) were retrieved after clinically indicated testing was completed, divided into aliquots (minimum 250 microliters each) and frozen at < −20°C for further testing. Available laboratory testing at CDC included testing for SARS-CoV-2 antibodies, molecular diagnostics for select respiratory pathogens, or SARS-CoV-2 whole genome sequencing of respiratory specimens with qualifying cycle threshold values. Specimens with a Ct value <33 were submitted for sequencing, and those with genome coverage >90% had a confirmed lineage reported. Results of research-use only laboratory tests were not expected to be provided to VAMC patients or their clinicians.

### Institutional Review and Consent

Modified protocols were reviewed and approved by the VAMC Research and Development Committee and institutional review boards for each of the five sites; in addition, the activity was reviewed by CDC and conducted consistent with applicable federal law and policy^3^. Sites tailored a generic consent form to provide their site-specific information and include necessary authorizations for release of medical records, for clinical specimen collection, and for potential future testing.

### Data Management

Standardized case report forms and data dictionaries were developed and used across all participating sites. Data were entered into a standardized surveillance database in REDCap, a secure web application for building and managing online surveys and databases designed to comply with Health Insurance Portability and Accountability Act (HIPAA) regulations (20, 21). Original participant medical records located at the participating sites were linked to surveillance data solely through sequentially assigned participant identification numbers. Local data were stored behind secure institutional firewalls. Following federal data handling requirements, data were transferred to CDC using participant identification numbers; personally identifiable information, such as patient name or medical record number, was not shared with CDC or other sites.

### Data Analysis

In addition to simple descriptive analyses of data from COVID-19 cases, including median and interquartile range (IQR), formal statistical comparisons of data from cases and controls may be performed to estimate pathogen-specific relative risk of disease progression and mortality. For example, to estimate the effects of underlying medical conditions on adverse clinical outcomes including mortality, respiratory failure, mechanical ventilation, intensive care unit (ICU) admission, and length of hospital stay, we calculated adjusted risk ratios (aRR) and 95% confidence intervals (CI) for these outcomes among participants with and without COVID-19 hospitalized from February 23–October 31, 2020.

## Results

Initial results from the adapted SUPERNOVA platform have been useful to demonstrate characteristics of adults hospitalized with COVID-19 and the spectrum of disease severity, clarify COVID-19 hospitalization rates among Veterans, and understand underlying medical conditions that increase risk of severe outcomes from COVID-19. As of March 4, 2021, SUPERNOVA included 1,235 patients with COVID-19 (COVID-19 cases) and 707 patients with other acute respiratory illnesses (primary controls) admitted to one of the five participating VAMC sites from February 23 through December 31, 2020. Overall, SUPERNOVA participants were predominantly male (93.1%), of non-Hispanic Black (45.8%) or non-Hispanic White (32.6%) race/ethnicity, and older, with a median age of 70 years (Table 2). Among 1,177 COVID-19 cases with available information about their hospitalization, many were severely ill: 332/1,177 (28.2%) were transferred to an intensive care unit (ICU); 107/1,137 (9.4%) received invasive mechanical ventilation; and 158/1,134 (13.9%) died while hospitalized.

**Table 2:** Characteristics of Veterans hospitalized with and without COVID-19—Surveillance Platform for Enteric and Respiratory Infectious Organisms at Veterans Affairs Medical Centers (SUPERNOVA), February–December 2020.

	**Veterans hospitalized with COVID-19 (cases)**	Veterans hospitalized without COVID-19 (primary controls)^*^
	***n* (%)**	***n* (%)**
**Total**	1,235	707
**City**
Atlanta, GA, USA	296 (24.0)	179 (25.3)
Bronx, NY, USA	220 (17.8)	101 (14.3)
Houston, TX, USA	285 (23.1)	194 (27.4)
Los Angeles, CA, USA	377 (30.5)	171 (24.2)
Palo Alto, CA, USA	57 (4.6)	62 (8.8)
**Median age** in years (IQR)	70 (59, 76)	69 (61, 75)
**Sex**
Male	1,153 (93.4)	656 (92.8)
Female	82 (6.6)	51 (7.2)
**Race/ethnicity**
Non-Hispanic Black	585 (47.4)	304 (43.0)
Non-Hispanic White	359 (29.1)	275 (38.9)
Hispanic or Latino	200 (16.2)	75 (10.6)
Asian	16 (1.3)	5 (0.7)
Native Hawaiian/Pacific Islander	10 (0.8)	7 (1.0)
American Indian/Alaskan Native	8 (0.7)	3 (0.4)
All other races^**^	57 (4.6)	38 (5.4)

*IQR, interquartile range*.

* *Primary controls had both a negative SARS-CoV-2 molecular test result and ≥1 acute respiratory infection (ARI) symptom*.

** *Includes multiple races (n = 3) and unknown race and/or ethnicity (n = 54)*.

To assess disparities in COVID-19 hospitalization rates by race/ethnicity, case data from SUPERNOVA were analyzed rapidly early in the pandemic. Among the first 621 laboratory-confirmed, hospitalized COVID-19 cases participating in SUPERNOVA, hospitalization rates were highest among Veterans aged ≥85 years (430 per 100,000), of Hispanic or Latino ethnicity (317 per 100,000), and of non-Hispanic Black race (298 per 100,000). Additionally, Veterans aged ≥65 years had a 4.5-fold increased risk of death compared with younger Veterans (95% confidence interval: 2.4–8.6). A short report on these findings highlighted the need for targeted prevention and timely treatment of COVID-19, particularly for older adults (22).

To enhance the evidence base for underlying medical conditions including those with limited evidence to date, we conducted an interim analysis of SUPERNOVA data on comorbidities among COVID-19 cases and controls for outcomes indicating more severe COVID-19 illness. Among 1,254 symptomatic participants through October 2020, including 786 with COVID-19 and 468 without COVID-19, a high prevalence of underlying medical conditions was documented among all participants; median number of underlying medical conditions was four (IQR 2–6) and five (IQR 3–7) among participants with and without COVID-19, respectively. The most common underlying medical conditions among all participants were hypertension (71.4%), cardiovascular disease (53.7%), diabetes mellitus (45.3%), and chronic non-asthmatic lung disease (41.0%). Compared with controls, after adjusting for age, sex, and race/ethnicity, COVID-19 case-patients had significantly higher risk of mortality, respiratory failure, and invasive mechanical ventilation, and longer hospital stays; 11 categories of underlying medical conditions increased risk for at least one of these outcomes: cancer, recent chemotherapy, cerebrovascular disease, chronic kidney disease, chronic non-asthmatic lung disease, cardiovascular disease, smoking, and diabetes mellitus (data not shown). Risk of death was more than three times higher among participants with COVID-19 (18.1%) compared to those without COVID-19 (6.8%) (aRR 3.07 [95% CI: 2.13, 4.41]).

## Discussion

Data from the SUPERNOVA platform enhance COVID-19 surveillance among hospitalized Veterans and are useful to describe the burden and natural history of COVID-19 in this key population. The SUPERNOVA population includes many males, older adults, racial and ethnic minority groups, and people with chronic diseases and other underlying medical conditions, who thus may be disproportionately affected by severe COVID-19 disease outcomes. Strengths of the SUPERNOVA platform for COVID-19 surveillance include the ability to collect and integrate multiple types of data, including clinical and illness outcome information, and laboratory test results from respiratory and serum specimens, with formal comparison between cases and controls with similar hospitalization dates. Furthermore, patients can be followed longitudinally over time. SUPERNOVA data will be useful to address several additional scientific questions related to COVID-19.

### COVID-19 Vaccine Effectiveness

Vaccine effectiveness (VE) against COVID-19 hospitalization among Veterans may be estimated using data obtained from this surveillance system by comparing vaccination status between hospitalized adults with and without laboratory-confirmed SARS-CoV-2 infection. Using a modified, prospective, test-negative case-control design (23, 24) to identify COVID-19 cases and controls with COVID-like signs or symptoms identified through passive and active surveillance, VE can be calculated using multivariable logistic regression with case status as the outcome and vaccination status as the main predictor, adjusting for potential confounders (e.g., site, age, sex, race/ethnicity, and underlying health status), where estimated VE = (1–adjusted odds ratio^4^) ×100 (25). To address potential misclassification of controls, VE estimates will be calculated using both primary and secondary controls. SUPERNOVA is one of several surveillance platforms CDC is using to assess effectiveness of COVID-19 vaccines in various populations (26). SUPERNOVA data can be used to evaluate effectiveness of full or partial vaccination against COVID-19 as well as specific vaccine products, reflecting real-world use. Data from SUPERNOVA showed that, during February 1–August 6, 2021, VE of mRNA COVID-19 vaccines against COVID-19 hospitalization was 80% among adults aged ≥65 years compared with 95% among adults aged 18–64 years (27).

### Virus Variants

Genomic sequencing of specimens collected in SUPERNOVA may be used to identify emergence of novel respiratory pathogens such as SARS-CoV-2 variants, which are a cause of concern due to their potential to exacerbate the trajectory of the COVID-19 pandemic (28). Monitoring for variants, particularly among VA patients with multiple hospital admissions for COVID-19, may provide evidence regarding disease severity, potential reinfections, and variant influence on countermeasures including diagnostics, therapeutics, and vaccines. Among SUPERNOVA participants, Delta became the predominant SARS-CoV-2 variant in July 2021 (27).

### Immune Responses

SUPERNOVA data may be able to address whether antibodies to SARS-CoV-2 or other common coronaviruses might modulate COVID-19 illness severity, using serum representing a spectrum of disease from adults hospitalized with COVID-19.

### Multisystem Inflammatory Syndrome in Adults

Case series have been reported of Multisystem inflammatory syndrome in adults (MIS-A) associated with SARS-CoV-2 infection (29). Data on laboratory values and imaging studies from SUPERNOVA may be used to identify possible cases of MIS-A to target these cases for additional chart review and follow-up.

### Health Care Use

COVID-19 can result in late sequelae and prolonged illness (“long COVID,” or post-acute sequelae of SARS-CoV-2 infection), but relatively little is known about typical clinical course and health care use following hospital discharge for COVID-19 (30). SUPERNOVA data, which include information on disposition and health care use through at least 90 days following initial hospital discharge for both COVID-19 cases and controls, might help fill this knowledge gap.

Limitations to this surveillance approach include the reliance on current policy of universal screening for SARS-CoV-2 infection of inpatients at the participating sites to identify cases and controls; if these policies were to change, misclassification of COVID-19 cases and controls might be more likely. In addition, timeliness of reporting varies by pandemic phase and site; during pandemic surges in COVID-19 cases at respective sites, adjustments included prioritizing identification of cases rather than controls, and implementing flexible or extended deadlines for data entry and submission to CDC. Furthermore, although a test-negative design for assessing VE minimizes differences by healthcare seeking behavior, other COVID-19 risk mitigation measures could be relevant. Addition of an interview component could help identify differential behaviors associated with COVID-19 acquisition or transmission between cases and controls, such as compliance with non-pharmaceutical interventions (e.g., mask-wearing, physical distancing) or known close contact with a person with COVID-19 in the 14 days preceding symptom onset.

## Conclusion

In this methods paper, we describe the adaptation of an existing five-site sentinel surveillance platform for acute gastroenteritis and acute respiratory illness to monitor COVID-19 among hospitalized adult Veterans in the United States. The SUPERNOVA surveillance platform, comprising passive and active surveillance and including medical record reviews and clinical specimen collection, pivoted to address new public health priorities during the COVID-19 pandemic. Plans include dissemination of results of ongoing data analyses to inform program and policy decisions with continued attention and responsiveness to public health emergency needs.

## Data Availability Statement

The original contributions presented in the study are included in the article/supplementary material; further inquiries can be directed to the corresponding author.

## Ethics Statement

The studies involving human participants were reviewed and approved by VAMC Research and Development Committee and Institutional Review Boards for each of the five sites; in addition, the activity was reviewed by CDC and conducted consistent with applicable federal law and policy (see e.g., 45 C.F.R. part 46.102(l) (2), 21 C.F.R. part 56; 42 U.S.C. §241(d); 5 U.S.C. §552a; 44 U.S.C. §3501 et seq.). The patients/participants provided their written informed consent to participate in this study.

## SUPERNOVA COVID-19 Surveillance Group

Ghazal Ahmadi-Izadi, LaSara Bell, Joy Burnette, Rijalda Deovic, Lauren Epstein, Amy Hartley, Elena Morales, Taressa Sergent, Tehquin Tanner, and Alexis Whitmire: Atlanta VA Medical Center, Atlanta, GA, United States. Katherine Elliot, Ilda Graham, Diki Lama, Awilda Mero, Ismael Pena, Adrienne Perea, Guerry Anabelle Perez, Johane Simelane, Sarah Smith, Gabriela Tallin, and Amelia Tisi: James J. Peters VA Medical Center, Bronx, NY, United States. Alonso Arellano Lopez, Miguel Covarrubias Gonzalez, Rosalba Gomez Morones, Bashir Lengi, Dena Mansouri, Gilberto Rivera Dominguez, Mariana Vanoye Tamez, and Blanca Vargas. Michael E. DeBakey VA Medical Center, Houston, TX, United States. Babak Aryanfar, Ian Lee-Chang, Karen Evangelista, Matthew Goetz, Evan Goldin, Chan Jeong, Anthony Matolek, Chad Mendoza, and Aleksandra Poteshkina: VA Greater Los Angeles Healthcare System, Los Angeles, CA, United States. Madhuri Agrawal, Jessica Lopez, Jude Lopez, and Theresa Peters: VA Palo Alto Health Care System, Palo Alto, CA, United States. Meredith McMorrow, Steve L. Evener, Rebecca Dahl, Daoling Bi, Lindsay Kim, and Aron J. Hall: Centers for Disease Control and Prevention, Atlanta, GA, United States.

## Author Contributions

MP, CC, JC, EM, KB, and DS designed the study and drafted the protocol. VM, SB, MR-B, DB, MH, and CL-O revised and approved the final protocol and oversaw data collection. AK, MP, JC, EM, KB, and DS managed and analyzed the data. All authors contributed to the manuscript and approved the submitted version.

## Funding

This study was funded by the Centers for Disease Control and Prevention *via* a contract with the Foundation for Atlanta Veterans Education and Research, Inc. (contract number 75D30119C04211).

## Author Disclaimer

The findings and conclusions in this report are those of the authors and do not necessarily represent the official position of the Centers for Disease Control and Prevention, Department of Veterans Affairs or United States Government.

## Conflict of Interest

VM has received investigator-initiated research grants (to the institution) and consultation fees from Eli Lilly, Bayer, Gilead Sciences, and ViiV. The remaining authors declare that the research was conducted in the absence of any commercial or financial relationships that could be construed as a potential conflict of interest.

## Publisher's Note

All claims expressed in this article are solely those of the authors and do not necessarily represent those of their affiliated organizations, or those of the publisher, the editors and the reviewers. Any product that may be evaluated in this article, or claim that may be made by its manufacturer, is not guaranteed or endorsed by the publisher.

## Footnotes

^1^COVID-19-like signs or symptoms included fever, new or worsened cough, new or worsened shortness of breath, loss of taste or smell, increased sputum production, SpO_2_ on room air <94%, invasive or non-invasive ventilation requirement, and pulmonary findings on chest imaging consistent with pneumonia.

^2^Symptoms of acute respiratory illness included fever, chills, cough, shortness of breath, sinus congestion/runny nose, or sore throat.

^3^See e.g., 45 C.F.R. part 46.102(l) (2), 21 C.F.R. part 56; 42 U.S.C. §241(d); 5 U.S.C. §552a; 44 U.S.C. §3501 et seq.

^4^Odds ratio for case status among vaccinated persons compared with unvaccinated persons.
